# Association of Veterinary Third-Generation Cephalosporin Use with the Risk of Emergence of Extended-Spectrum-Cephalosporin Resistance in *Escherichia coli* from Dairy Cattle in Japan

**DOI:** 10.1371/journal.pone.0096101

**Published:** 2014-04-22

**Authors:** Toyotaka Sato, Torahiko Okubo, Masaru Usui, Shin-ichi Yokota, Satoshi Izumiyama, Yutaka Tamura

**Affiliations:** 1 Laboratory of Food Microbiology and Food Safety, Department of Health and Environmental Sciences, School of Veterinary Medicine, Rakuno Gakuen University, Ebetsu, Japan; 2 Department of Microbiology, Sapporo Medical University School of Medicine, Sapporo, Japan; 3 Nemuro District Agriculture Mutual Aid Association, Nakashibetsu, Japan; Institut National de la Recherche Agronomique, France

## Abstract

The use of extended-spectrum cephalosporins in food animals has been suggested to increase the risk of spread of *Enterobacteriaceae* carrying extended-spectrum β-lactamases to humans. However, evidence that selection of extended-spectrum cephalosporin–resistant bacteria owing to the actual veterinary use of these drugs according to criteria established in cattle has not been demonstrated. In this study, we investigated the natural occurrence of cephalosporin-resistant *Escherichia coli* in dairy cattle following clinical application of ceftiofur. *E. coli* isolates were obtained from rectal samples of treated and untreated cattle (n = 20/group) cultured on deoxycholate-hydrogen sulfide-lactose agar in the presence or absence of ceftiofur. Eleven cefazoline-resistant isolates were obtained from two of the ceftiofur-treated cattle; no cefazoline-resistant isolates were found in untreated cattle. The cefazoline-resistant isolates had mutations in the chromosomal *ampC* promoter region and remained susceptible to ceftiofur. Eighteen extended-spectrum cephalosporin–resistant isolates from two ceftiofur-treated cows were obtained on ceftiofur-supplemented agar; no extended-spectrum cephalosporin–resistant isolates were obtained from untreated cattle. These extended-spectrum cephalosporin–resistant isolates possessed plasmid-mediated β-lactamase genes, including *bla*_CTX-M-2_ (9 isolates), *bla*_CTX-M-14_ (8 isolates), or *bla*_CMY-2_ (1 isolate); isolates possessing *bla*_CTX-M-2_ and *bla*_CTX-M-14_ were clonally related. These genes were located on self-transmissible plasmids. Our results suggest that appropriate veterinary use of ceftiofur did not trigger growth extended-spectrum cephalosporin–resistant *E. coli* in the bovine rectal flora; however, ceftiofur selection *in vitro* suggested that additional ceftiofur exposure enhanced selection for specific extended-spectrum cephalosporin–resistant β-lactamase-expressing *E. coli* clones

## Introduction

β-Lactam antimicrobials are used worldwide in clinical settings. Extended-spectrum cephalosporins (ESCs; third- and fourth-generation cephalosporins such as cefpodoxime [CPD], ceftazidime [CAZ], and cefepime [FEP]) are broad-spectrum antimicrobials that have been listed by the World Health Organization (WHO) as critically important for human health [1]. However, the clinical occurrence of ESC-resistant *Enterobacteriaceae* has increased [3], [4]. Numerous bacterial infections in food-producing animals and humans are treated with first- or second-generation cephalosporins such as cefazoline (CFZ), cefalexin (LEX), and cefuroxime (CXM), and ESCs such as ceftiofur (CTF) and CPD. In the WHO ranking of antimicrobials according to their importance in human medicine, the ESCs that are also used in veterinary medicine are listed at the highest rank (critically important antimicrobial agents) on the basis of 2 criteria: (1) the agent or class is the sole therapeutic option or one of few alternatives available to treat serious human disease; and (2) the antimicrobial agent or class is used to treat diseases caused by organisms that may be transmitted via nonhuman sources or diseases caused by organisms that may acquire resistance genes from nonhuman sources [5]. ESCs used in humans and animals are of the same general class and share the same mode of action, even if they differ chemically [6]. Thus, the appearance of ESC-resistant bacteria can be attributable to mechanisms common between humans and animals (mainly by the acquisition of extended-spectrum β-lactamase genes and AmpC-type β-lactamase genes such as *bla*_CTX-M_ and *bla*_CMY_), and interspecies transmission of ESC-resistant bacteria can occur [7], [8]. ESC-resistant *Enterobacteriaceae* are found in food animals and their products [7], [9]–[12]. Therefore, discussing issues related to the use of ESCs in veterinary medicine necessitates scientific evidence regarding the joint role played by ESCs in human and veterinary medicine.

CTF is a third-generation cephalosporin that is commonly used in veterinary medicine worldwide [13]–[15]. In Japan, CTF has been approved for use in cattle as a second-line drug for the treatment of pneumonia and as a first-line drug in serious infectious diseases in dairy cattle. *E. coli* is a commensal bacterial species in cattle feces [16]; some *E. coli* strains act as enteric pathogens in humans and/or are resistant to antimicrobials. [17]. Therefore, it is essential to characterize the *E. coli* with naturally occurring ESC resistance in bovine rectal flora because of the veterinary use of CTF, which may select for antimicrobial resistance. Previous studies have suggested an association between CTF use and the occurrence of ESC-resistant *E. coli* in cattle [2], [13]–[15]. However, it is not known if *E. coli* with naturally occurring ESC resistance is selected for by appropriate veterinary ECS use because many studies have involved artificial intragastric inoculation with extended-spectrum β-lactamase–producing *E. coli* mutants and most do not record antimicrobial use or clinical criteria, such as, detail methods for ECS use, dose, or washout for every cow. All of these factors are important to consider when evaluating for a causal relationship between ECS use and naturally occurring ECS resistance.

In this study, to evaluate the risk of selection of ESC-resistant bacteria related to veterinary treatment with a suitable third-generation cephalosporin, we tried to isolate *E. coli* with naturally occurring ESC resistance from the rectal flora of CTF-treated or untreated dairy cattle after the washout period.

## Materials and Methods

### Bacterial Samples

We collected 20 dairy bovine rectal feces samples from dairy cattle treated with Excenel (cows 1–20; CTF sodium injection; Pfizer, New York City, USA). Ethical authority was not required according to the Epidemiological and Animal Ethical Research Committee of Rakuno Gakuen University because CTF treatment in this case was performed as part of general clinical treatment, compliant with the Veterinarians Acts and the Pharmaceutical Affairs Law defined by the Ministry of Agriculture, Forestry, and Fisheries in Japan. Briefly, cattle received intramuscular injections of 1–2 mg·kg^−1^·day^−1^ CTF for 3 days for serious infectious diseases such as refractory pneumonia, puerperal fever, and hoof disease. This represented the first CTF treatment for these animals, and none had received any other antimicrobials for at least 3 months before sampling. Rectal feces samples were collected after the 8-day washout period, at which point the remaining CTF concentration in the organs and products have no effect on human health, according to the Pharmaceutical Affairs Law defined by the Ministry of Agriculture, Forestry, and Fisheries. The untreated controls included 20 dairy cattle (numbers 21–40) that did not receive any other antimicrobials for at least 3 months and no CTF use for at least 1.5 years before sampling. We did not sample non-treated cattle from herds that contain CTF-treated cattle. All samples were collected from independent farms in Betsukai (Hokkaido, Japan), the most productive dairying area in Japan (at least 100 cattle per farm). We had permission from the farms to collect fecal matter from their private property and CTF use in this study.

### Isolation of *E. coli*

Fecal samples (1 g) were dissolved in 9 mL of 0.85% sterile saline solution; 100 µL was immediately spread on deoxycholate-hydrogen sulfide-lactose (DHL) agar (Nissui, Tokyo, Japan) supplemented with 4 µg/mL CTF and incubated for 24 h at 37°C. CTF-free DHL plates served as controls. Samples were subcultured on nutrient agar (Nissui) at a maximum of 10 colonies per agar plate. The biochemical properties of these colonies were examined using triple sugar iron medium (Nissui), lysine indole motility medium (Nissui), and oxidase tests. Final identification of *E. coli* was performed by API20E (bioMérieux, Tokyo, Japan).

### Susceptibility Testing

β-Lactam resistance was screened using CFZ KB-disks (Eiken, Tokyo, Japan) according to the manufacturer’s instructions. Isolates showing resistance to CFZ in the KB-disk method were assessed to determine the minimum inhibitory concentration (MIC) by using the microdilution method according to the recommendations of the Clinical and Laboratory Standards Institute (CLSI; 2008) [18]. MICs were determined for eight β-lactam antimicrobials: AMP, CFZ, LEX, CXM, CTF, CPD, CAZ, FEP, and two mixtures of clavulanic acid (CVA) (CVA/AMP and CVA/CTF). Breakpoint values were defined according to 2008 CLSI recommendations, except in the case of LEX, CXM, CAZ, and FEP, which were defined according to 2011 CLSI recommendations [19], because the break points for these agents have not been defined for veterinary pathogens. Antimicrobial plates for microdilution testing were purchased from Eiken.

### Detection of β-lactamase Genes

β-Lactamase genes were identified by PCR and direct DNA sequencing. *bla*_CTX-M_ was detected as described by Xu et al. [20], and plasmid-mediated *ampC* was detected as described by Pérez-Pérez et al. [21]. The presence of *bla*_TEM_, *bla*_SHV_, and mutations in the chromosomal *ampC* promoter region was detected according to Kojima et al. [11]. Nucleotide sequences were determined with a BigDye Terminator v3.1 Cycle Sequencing Kit and a 3130 Genetic Analyzer (Applied Biosystems, Foster City, CA).

### Pulsed-field Gel Electrophoresis (PFGE)

PFGE was performed according to the method outlined by PulseNet USA [22] by using *Xba*I (Takara-Bio, Tokyo, Japan). The CHEF-DR III system (Bio-Rad Laboratories, Hercules, CA, USA) was used with the following running conditions: 19 h at 11.3°C, voltage of 6 V, ramped with an initial forward time of 2.2 s, and a final forward time of 54.2 s. After electrophoresis, gels were stained with ethidium bromide and photographed. The banding patterns were visually interpreted using published guidelines, and Dice similarity indices were calculated by cluster analysis.

### Transferability Test of β-lactamase Genes

Broth-mating experiments were performed using rifampicin-resistant ML4909 (F^−^ *galK2 galT22 hsdR metB1 relA supE44* rifampicin-resistant) as a recipient strain [21]. Donors and recipients were grown in tryptic soy broth (TSB, Nissui) to the logarithmic phase; they were then mixed in a total volume of 2 mL at a 1∶9 (v/v) ratio, and 2 mL fresh TSB was added. The mating cultures were incubated overnight at 37°C. Transconjugants were selected on CTF (final concentration, 4 µg/mL)- and rifampicin (final concentration, 64 µg/mL)-containing MH agar.

### Plasmid Profiling and Southern Hybridization Analysis

Plasmid profiling was performed according to previously described methods [24]. Plasmid incompatibility (Inc) groups were determined by PCR with the following primers: HI1, HI2, I1-Iγ, X, L/M, N, FIA, FIB, W, Y, P, FIC, A/C, T, FIIAs, F, K, and B/O [25].

Southern hybridization was performed as follows. Probes were prepared by PCR. Probes for *bla*_CTX-M-2_ and *bla*_CTX-M-14_ were prepared using a CTX-M consensus primer set [26]. The probe for *bla*_CMY-2_ was prepared using primers described by Pérez-Pérez and Hanson [21]. These PCR products were labeled using a PCR DIG Labeling Mix (Roche Diagnostic, Tokyo, Japan) according to the manufacturer’s instructions. Plasmid DNA was separated by 0.8% (w/v) agarose gel electrophoresis at 100 V for 70 min. The DNA in the gel was transferred to a positive membrane (Roche Diagnostics) by the capillary method. Pre-hybridization (>30 min) and hybridization (>16 h) were performed using Easy Hyb solution (Roche Diagnostics) under high-stringency conditions, and digoxigenin (DIG) in the hybrids was detected using a DIG Luminescent Detection Kit (Roche Diagnostics) according to the manufacturer’s instructions. A hyper MP film (GE Healthcare Japan, Tokyo, Japan) was exposed to the membranes for 2 min at room temperature and developed in a Kodak X-Omat processor.

### Statistical Analysis

Statistical significance was determined using chi-square test and Fisher’s exact tests. Significance was set at *p*<0.05.

## Results

### Isolation and Antimicrobial Resistance of *E. coli*

#### Using non-supplemented agar

In this study, 193 and 182 *E. coli* isolates were obtained from CTF-treated and untreated cattle, respectively. We screened for CFZ resistance by the disk diffusion method. From the 193 strains isolated from CTF-treated cattle, 11 isolates resistant to CFZ were obtained; however, no CFZ-resistant *E. coli* was obtained from untreated cattle (*p*<0.05). The 11 CFZ-resistant isolates (from cow No. 5 [10 strains] and cow No. 7 [1 strain]) were also resistant to AMP and CXM, but not CTF; CVA did not affect the MICs of AMP or CTF (Table 1). All 11 isolates carried mutations in the chromosomal *ampC* promoter region.

**Table 1:** β-lactam antimicrobial susceptibilities and detection of β-lactamase genes in AMP-resistant isolates in non-supplemented agar.

Cow number	Number of strains/tested colonies	CTF treatment	MIC (µg/mL)						β-lactamase gene
			AMP(≥32)^a^	AMP/CVA	CFZ(≥32)	CXM(≥32)	CTF(≥8)	CTF/CVA	
5	10/10	+	>128	64/32	64–128	32	1	1/0.5	−1(CtoT)/−18(GtoA)/−42(CtoT)/−82(AtoG)*
7	1/10	+	>128	64/32	128	32	2	2/1	−1(CtoT)/−18(GtoA)/−42(CtoT)/−82(AtoG)*

a Breakpoint; *Mutations in the chromosomal *ampC* promoter region.

#### Using CTF-supplemented agar

Eighteen *E. coli* isolates were obtained on CTF-supplemented agar (from CTF-treated cow No. 7 [10 strains] and cow No. 13 [8 strains]); no resistant strains were isolated from the untreated group (*p*<0.05). Seventeen isolates were resistant to AMP, CFZ, LEX, CXM, CPD, and CTF, and CVA influenced the MICs of AMP and CTF (Table 2). The *E. coli* isolates showed CTF resistance from cow nos. 7 and 13 possessed *bla*_CTX-M-2_ and *bla*_CTX-M-14_, respectively. The last strain from CTF-treated cow No. 7 showed resistance to AMP, CFZ, LEX, CXM, CPD, CAZ, and CTF; however, CVA did not affect the MICs of AMP and CTF in this isolate. This strain possessed *bla*_CMY-2_. None of the isolates exceeded the breakpoint of FEP (Tables 1 and 2).

**Table 2:** β-Lactam susceptibilities and detection of β-lactamase genes in cephalosporin-resistant isolates in CTF-supplemented agar.

Cow number	Number of strains	CTF treatment	MIC (µg/mL)										Inc. type	β-lactamase gene
			AMP (≥32)^a^	AMP/CVA	CFZ (≥32)	LEX (≥32)	CXM (≥32)	CTF (≥8)	CTF/CVA	CPD (≥8)	CAZ (≥16)	FEP (≥32)		
7	9	+	>128	8/4	>128	>128	>128	>32	1/0.5	>128	2	16	N, FIA, FIB	*bla*_CTX-M-2_
7	1	+	128	64/32	>128	32	32	8	8/4	>128	32	≤0.125	I1-Iγ, FIB	*bla*_CMY-2_
13	8	+	>128	8/4	>128	>128	>128	>32	1/0.5	>128	1–2	4–8	I1-Iγ	*bla*_CTX-M-14_

a Break point.

### PFGE Analysis and Plasmid Analysis

**Figure 1:**
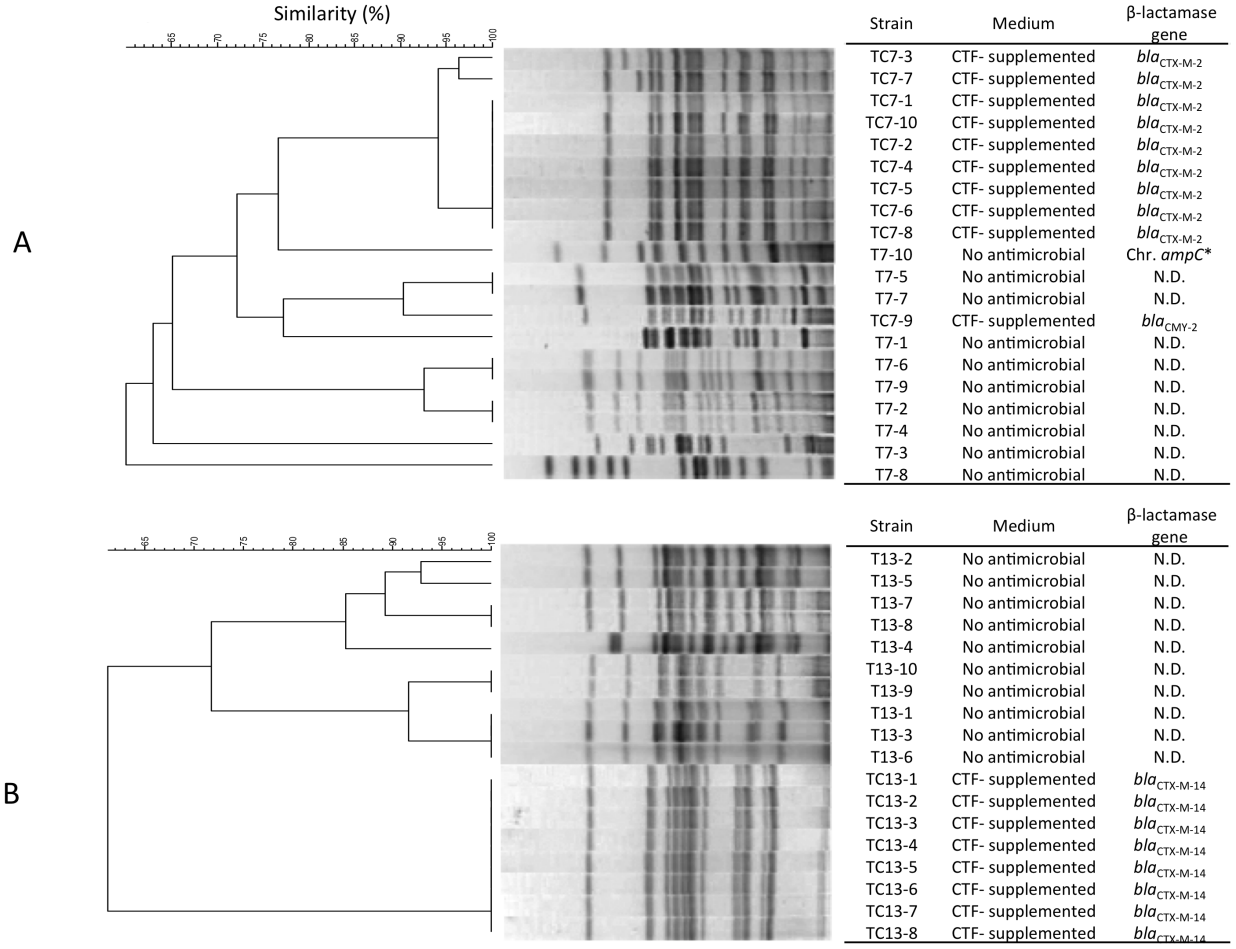
Pulsed-field gel electrophoresis of CTF-resistant *E. coli* isolates from two CTF-treated cattle. A, PFGE analysis of 20 isolates obtained from cow No.

**Figure 2:**
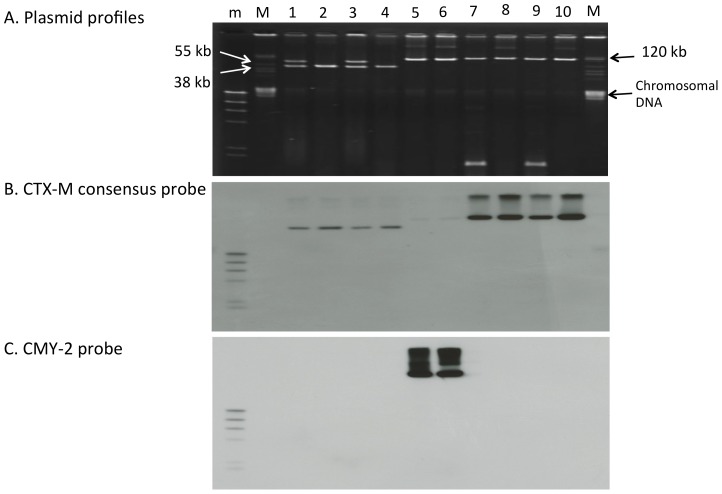
Plasmid profiling and Southern hybridization of β-lactamase genes in *E. coli* isolates from CTF-treated cattle. A, Plasmid profiling. B, Southern hybridization of the *bla*_CTX-M_ consensus probe. C, Southern hybridization of the *bla*_CMY-2_ probe. Lane1, ML4909 (recipient); lane 2, TC7-1 (possesses *bla*_CTX-M-2_); lane 3, TcTC7-1; lane 4, TC7-2 (possesses *bla*_CTX-M-2_); lane 5, TcTC7-2; lane 6, TC7-9 (possesses *bla*_CMY-2_); lane 7, TcTC7-9; lane 8, TC13-1 (possesses *bla*_CTX-M-14_); lane 9, TcTC13-1; lane 10, TC13-2 (possesses *bla*_CTX-M-14_); lane 11, TcTC13-2; m, DNA Molecular Weight Marker II, DIG-labeled; M, BAC-Tracker Supercoiled DNA Ladder. *bla*_CTX-M-2_ and *bla*_CTX-M-14_ were detected using a CTX-M consensus probe.

To determine the clonal relationship of isolates exhibiting differential CTF selection properties, we performed PFGE of isolates derived from non-supplemented and CTF-supplemented agar after collection from two CTF-treated cattle (Nos. 7 and 13; Figure 1). The PFGE pattern showed that these isolates were clearly different clones. Isolates harboring *bla*_CTX-M-2_ and *bla*_CTX-M-14_, which were isolated from CTF-supplemented agar, exhibited mostly identical PFGE patterns. Plasmid profiling also showed that strains isolated from CTF-supplemented agar possessed identically sized plasmid(s) harboring their respective β-lactamase gene types (Figure 2). These results indicated that resistant strains carrying *bla*_CTX-M-2_ from cow No. 7 and those carrying *bla*_CTX-M-14_ from cow no. 13 originated from a single clone in each cow.

### Transferability Test and Southern Hybridization of β-lactamase Genes

We investigated the transferability of β-lactamase genes using recipient ML4909 cells. The donors were TC7-1 and TC7-2, which possessed *bla*_CTX-M-2_ (isolated from cow No. 7); TC7-9, which possessed *bla*_CMY-2_ (isolated from cow No. 7); and TC13-1 and TC13-2, which possessed *bla*_CTX-M-14_ (isolated from cow No. 13). All detected β-lactamase genes could be transferred to the recipient, and the MICs of the transconjugants increased at a level similar to that of the donor (Table 3). Replicon typing and Southern hybridization showed that *bla*_CTX-M-2_ was located in an IncN plasmid (about 40 kb), *bla*_CMY-2_ was located in I1-Iγ and/or FIB plasmids (more than 100 kb), and *bla*_CTX-M-14_ was located in an I1-Iγ plasmid (more than 100 kb; Table 3 and Figure 2).

**Table 3:** β-Lactam susceptibilities and detection of β-lactamase genes in transconjugants from CTF-resistant isolates.

Strain	Characteristic	MIC (µg/mL)						Inc. group	β-lactamase gene
		AMP	AMP/CVA	CFZ	CXM	CTF	CTF/CVA		
ML4909	Recipient	4	2/1	2	<1	<0.5	<0.5/0.25	ND	ND^a)^
TC7-1	Donor	>128	8/4	>128	>128	>32	1/0.5	N, FIA, FIB	*bla*_CTX-M-2_
TcTC7-1	Transconjugant	>128	8/4	>128	>128	>32	1/0.5	N	*bla*_CTX-M-2_
TC7-2	Donor	>128	8/4	>128	>128	>32	1/0.5	N, FIA, FIB	*bla*_CTX-M-2_
TcTC7-2	Transconjugant	>128	8/4	>128	>128	>32	1/0.5	N	*bla*_CTX-M-2_
TC7-9	Donor	128	64/32	>128	32	8	8/4	I1-Iγ, FIB	*bla*_CMY-2_
TcTC7-9	Transconjugant	64	64/32	128	8	8	4/2	I1-Iγ, FIB	*bla*_CMY-2_
TC13-1	Donor	>128	8/4	>128	>128	>32	1/0.5	I1-Iγ	*bla*_CTX-M-14_
TcTC13-1	Transconjugant	>128	8/4	>128	>128	>32	<0.5/0.25	I1-Iγ	*bla*_CTX-M-14_
TC13-2	Donor	>128	8/4	>128	>128	>32	1/0.5	I1-Iγ	*bla*_CTX-M-14_
TcTC13-2	Transconjugant	>128	4/2	>128	>128	>32	<0.5/0.25	I1-Iγ	*bla*_CTX-M-14_

a ND, not detected.

## Discussion

The occurrence of antimicrobial-resistant bacteria, including cephalosporin-resistant bacteria, is thought to be related to selection pressures resulting from antimicrobial consumption [27]–[29]. Previous studies have suggested an association between CTF use and the occurrence of ESC-resistant *E. coli* in cattle [2], [13]–[15]. Although it has not reported an association between CTF use and the occurrence of ESC-resistant *E. coli* in cattle in Japan, a previous study showed that 6 (1.5%) of 396 *E. coli* isolates obtained from bovine fecal samples in Japan showed ESC resistance [30]. These data indicate an association between the isolation of ESC-resistant *E. coli* and ESC use in Japan. However, evidence supporting this association is lacking because the histories of clinical CTF use and compliance with clinical criteria were unknown and a cohort study on veterinary CTF use has never been performed. Thus, the estimation of emergence of ESC-resistant *E. coli* due to suitable clinical ESC use could help re-evaluate antimicrobial therapy to avoid the spread of ESC-resistant bacteria.

CTF was used to treat refractory pneumonia and other serious infectious diseases such as puerperal fever and hoof disease in dairy cattle, according to our inquiry survey. In this study, cephalosporin-resistant isolates were found only in CTF-treated animals. All of these isolates possessed mutations in chromosomal *ampC* and were resistant to AMP and first- and second-generation cephalosporins (CFZ and CXM), but not CTF. Thus, we conclude that if CTF is used appropriately (1–2 mg·kg^−1^·day^−1^ for 3 days) in Japanese veterinary practice, washout periods will increase the frequency of naturally occurring first- and second-generation cephalosporin resistance in *E. coli*, but will not influence the natural occurrence of ESC-resistant *E. coli* in dairy cattle.

A previous study reported the isolation of ESC-resistant *E. coli* (possessing *bla*_CMY-2_) from fecal samples of calves on 8 µg/mL CTF-supplemented agar, but not on non-supplemented agar [14]. This finding suggests that ESC-resistant *E. coli* are present in the bovine rectal flora at low frequency, and additional CTF exposure selects for these ESC-resistant *E. coli*. However, the applicability of this result to real-world CTF treatment in dairying is unknown, because the histories of clinical CTF use (or other β-lactams) in these calves were unknown. In this study, all CTF-resistant isolates were obtained from treated cattle and after culture on CTF-supplemented agar; these isolates possessed a plasmid-encoded β-lactamase gene, *bla*_CTX-M-2_, *bla*_CTX-M-14_, or *bla*_CMY-2_. Importantly, PFGE analysis showed that although ESC-resistant clones were not yet predominant in the rectal flora at the end of the washout period after CTF treatment, *in vitro* CTF exposure led to the selection of specific ESC-resistant clones in CTF-treated cattle. However, we could not determine which CTF dose (1 or 2 mg/kg of CTF) was high risk in terms of selection of ECS-resistant *E. coli* by *in vitro* testing because these dose were scattered regardless of whether ECS-resistant *E. coli* were isolated or not. Therefore, these results suggest that although appropriate CTF use (1–2 mg·kg^−1^·day^−1^ for 3 days) in Japanese veterinary practice dose not influence the natural occurrence of ESC-resistant *E. coli* in cattle, if CTF is used inappropriately, such as by overuse and/or subcutaneous use, it might encourage the selection and spread of broad-spectrum cephalosporin-resistant *E. coli* clones in bovine flora as suggested by *in vitro* CTF selection.

β-Lactamase genes in ESC-resistant isolates were located on Inc-type plasmids, i.e., *bla*_CTX-M-2_ (Inc-N), *bla*_CTX-M-14_ (Inc-I1-Iγ), and *bla*_CMY-2_ (Inc_-_I1-Iγ and/or FIB), and all were capable of self-transmission. *bla*_CTX-M2_ was found in *E. coli* from cattle in Japan from 2000 to 2001 [30]. *bla*_CMY-2_ was found in I1-Iγ and A/C plasmids in *Salmonella enterica* serovar Typhimurium isolated from cattle in Japan in 2007 [31]. *bla*_CTX-M-14_ was found in *S. enterica* serovar Enteritidis from chicken meat imported from China and sold by a retailer in Japan in 2004 [32]. The presence of these genes suggests β-lactamase genes producing ESC resistance are already widespread in Japanese livestock and their products. Although these data do not show an association between veterinary ESC use and the presence of β-lactamase genes in ESC-resistant *E. coli*, the presence of these genes might be selected for by cephalosporin use or co-selected by other antimicrobials used in veterinary medicine.

Furthermore, the ESC-resistant *E. coli* isolated in current study showed resistance to both ESCs used in veterinary medicine and to ESCs used in human medicine, and their transferable β-lactamase genes have been detected in humans in a variety of clinical settings worldwide [33]. Remarkably, *bla*_CTX-M-2_ and *bla*_CTX-M-14_ have also been found in *E. coli* isolates from humans in Japan (*bla*_CTX-M-2_ in Inc-N and *bla*_CTX-M-14_ in Inc-Il plasmids [4], [34], similar to the pattern observed in our study). In particular, *E. coli* O25 (undetermined H-antigen)-ST131, which frequently possesses *bla*_CTX-M-14_, is the most common strain that spreads to humans [34]. Our study and other studies suggest that human health may be at increased risk from the overuse of cephalosporins in livestock and that further genetic and epidemiological investigations are required to determine whether there is direct transmission of ESC-resistant *E. coli* and their β-lactamase genes from livestock to humans.

In conclusion, although appropriate veterinary use of a third-generation cephalosporin, CTF, increased the occurrence of first- and second-generation cephalosporin-resistant *E. coli,* it did not influence the natural occurrence of ECS-resistant *E. coli* in dairy cattle. However, *in vitro* CTF selection suggested that inappropriate CTF use in veterinary practice might increase the risk of selection of ESC-resistant *E. coli* possessing *bla*_CTX-M-2_, *bla*_CMY-2_, or *bla*_CTX-M-14_. Therefore, veterinary use of ESCs should be carefully monitored and used appropriately as described by the Joint FAO/WHO/OIE Expert Meeting on Critically Important Antimicrobials [6] to prevent the spread of ESC-resistant bacteria in veterinary medicine.
